# Repeated intermittent administration of (*R*)-ketamine during juvenile and adolescent stages prevents schizophrenia-relevant phenotypes in adult offspring after maternal immune activation: a role of TrkB signaling

**DOI:** 10.1007/s00406-021-01365-6

**Published:** 2022-01-03

**Authors:** Yunfei Tan, Yuko Fujita, Yaoyu Pu, Lijia Chang, Youge Qu, Xinming Wang, Kenji Hashimoto

**Affiliations:** Division of Clinical Neuroscience, Chiba University Center for Forensic Mental Health, Chiba, 260-8670 Japan

**Keywords:** Cognitive deficits, Psychosis, Prevention, (*R*)-ketamine, TrkB

## Abstract

Maternal immune activation (MIA) plays a role in the etiology of schizophrenia. MIA by prenatal exposure of polyinosinic:polycytidylic acid [poly(I:C)] in rodents caused behavioral and neurobiological changes relevant to schizophrenia in adult offspring. We investigated whether the novel antidepressant (*R*)-ketamine could prevent the development of psychosis-like phenotypes in adult offspring after MIA. We examined the effects of (*R*)-ketamine (10 mg/kg/day, twice weekly for 4 weeks) during juvenile and adolescent stages (P28–P56) on the development of cognitive deficits, loss of parvalbumin (PV)-immunoreactivity in the medial prefrontal cortex (mPFC), and decreased dendritic spine density in the mPFC and hippocampus from adult offspring after prenatal poly(I:C) exposure. Furthermore, we examined the role of TrkB in the prophylactic effects of (*R*)-ketamine. Repeated intermittent administration of (*R*)-ketamine during juvenile and adolescent stages significantly blocked the development of cognitive deficits, reduced PV-immunoreactivity in the prelimbic (PrL) of mPFC, and decreased dendritic spine density in the PrL of mPFC, CA3 and dentate gyrus of the hippocampus from adult offspring after prenatal poly(I:C) exposure. Furthermore, pretreatment with ANA-12 (TrkB antagonist: twice weekly for 4 weeks) significantly blocked the beneficial effects of (*R*)-ketamine on cognitive deficits of adult offspring after prenatal poly(I:C) exposure. These data suggest that repeated intermittent administration of (*R*)-ketamine during juvenile and adolescent stages could prevent the development of psychosis in adult offspring after MIA. Therefore, (*R*)-ketamine would be a potential prophylactic drug for young subjects with high-risk for psychosis.

## Introduction

Schizophrenia is a complex and heterogeneous psychiatric disorder that has significant economic, clinical and social impacts worldwide. The range of symptoms of schizophrenia includes positive symptoms (i.e., delusions, hallucinations, thinking disorders), negative symptoms (i.e., anhedonia, depression, social withdrawal, flawed thinking), and cognitive impairment (i.e., attention, memory, processing speed). Cognitive impairment is the core symptom in patients with schizophrenia that is present across the course of the illness [1–3]. Increasing evidence shows that cognitive impairment is detected in childhood and adolescence prior to the onset of psychosis [4–6]. A meta-analysis shows that cognitive impairment in people at clinical high risk for psychosis who converted to psychosis was greater than in those who did not convert to psychosis [7]. Collectively, it is important to treat cognitive impairment in young subjects with ultra-high risk for psychosis [8].

Multiple epidemiological studies suggest that maternal immune activation (MIA) such as viral infection during pregnancy plays a role in the etiology of schizophrenia [9–11]. MIA using polyriboinosinic-polyribocytidilic acid [poly(I:C)], a Toll-like receptor 3 agonist, has been widely used as an animal model for schizophrenia since adult offspring after prenatal poly(I:C) exposure show behavioral abnormalities relevant to schizophrenia [12–17]. Interestingly, cognitive impairment is detected before the onset of schizophrenia, suggesting the potential benefit of early intervention in the prodromal symptoms of young adults with a high risk for psychosis [8]. We previously found cognitive deficits of juvenile offspring from poly(I:C)-treated pregnant mice, suggesting that juvenile offspring after MIA may be a prodromal stage for schizophrenia [18–21].

(*R,S*)-ketamine, an *N*-methyl-d-aspartate receptor (NMDAR) antagonist, is an equal mixture of (*R*)-ketamine and (*S*)-ketamine. We demonstrated that (*R*)-ketamine, enantiomer of (*R,S*)-ketamine, has greater potency and longer-lasting antidepressant-like effects than (*S*)-ketamine in animal models of depression despite lower affinity at NMDAR [22–28]. Interestingly, side effects of (*R*)-ketamine in rodents, monkey and humans were less than (*R,S*)-ketamine and (*S*)-ketamine [23, 29–35]. Collectively, (*R*)-ketamine would be a novel antidepressant without detrimental side effects of (*R,S*)-ketamine [36–41].

The NMDAR antagonist phencyclidine (PCP) has been widely used as an animal model of schizophrenia since PCP caused schizophrenia-like symptoms in humans [42–45]. We reported that PCP-induced cognitive deficits in mice were ameliorated by subsequent repeated intermittent administration of (*R*)-ketamine, but not (*S*)-ketamine [46]. Furthermore, ANA-12 (TrkB antagonist) blocked the beneficial effects of (*R*)-ketamine in PCP model [46], suggesting a role of TrkB in the beneficial effects of (*R*)-ketamine. However, there are no reports investigating the effect of (*R*)-ketamine in MIA model.

In this study, we investigated whether repeated intermittent administration of (*R*)-ketamine during juvenile and adolescent stages could prevent the development of cognitive deficits, reduced immunoreactivity of parvalbumin (PV) and dendritic spine density in the medial prefrontal cortex (mPFC) in adult offspring after maternal poly(I:C) exposure. Furthermore, we examined the effects of ANA-12 in the beneficial effects of (*R*)-ketamine in the MIA model since brain-derived neurotrophic factor (BDNF) and its receptor TrkB signaling could play a role in the pharmacological effects of (*R*)-ketamine [23, 46–50].

## Methods and materials

### Animals and poly(I:C) injection

Pregnant female mice ddY (5 days) were purchased from SLC Company Japan (Hamamatsu, Shizuoka, Japan). The pregnant mice were intraperitoneally (i.p.) injected with poly(I:C) (5.0 mg/kg/day from E12 to E17) or an equivalent volume of saline as previously reported [18–21]. The offspring were separated from their mother mice at 3-weeks old, and male mice were kept in cages in groups of three to five. The mice were housed in a 22.5 × 33.8 × 14.0 cm transparent Polycarbonate cage, at a room temperature of 23 ± 1 °C, a humidity of 55 ± 5%, and the controlled light–dark cycle was 12/12 h (lights on from 07:00 a.m. to 07:00 p.m.), with ad libitum food (CE-2; CLEA Japan, Inc., Tokyo, Japan) and water. All experiments were performed in accordance with the animal experiment guidelines of Chiba University. The protocol (permission number: 1–331 and 2–131) was approved by the Institutional Animal Care and Use Committee of Chiba University.

### Drugs

Poly(I:C) was purchased from CALBIOCHEM (San Diego, CA, USA). (*R*)-ketamine hydrochloride was prepared in our laboratory, as previously reported [22]. ANA-12 (N-[2-[[(Hexahydro-2-oxo-1H-azepin-3-yl)amino]carbonyl]phenyl]-benzo[b]thiophene-2-carboxamide: 0.5 mg/kg) (Sigma-Aldrich Japan, Tokyo, Japan) was dissolved in phosphate-buffered saline (PBS) containing 17% dimethyl sulfoxide (DMSO) as previously reported [25, 46, 47, 51–54]. Other drugs are purchased from commercial sources.

### Drug administration

**Fig. 1:**
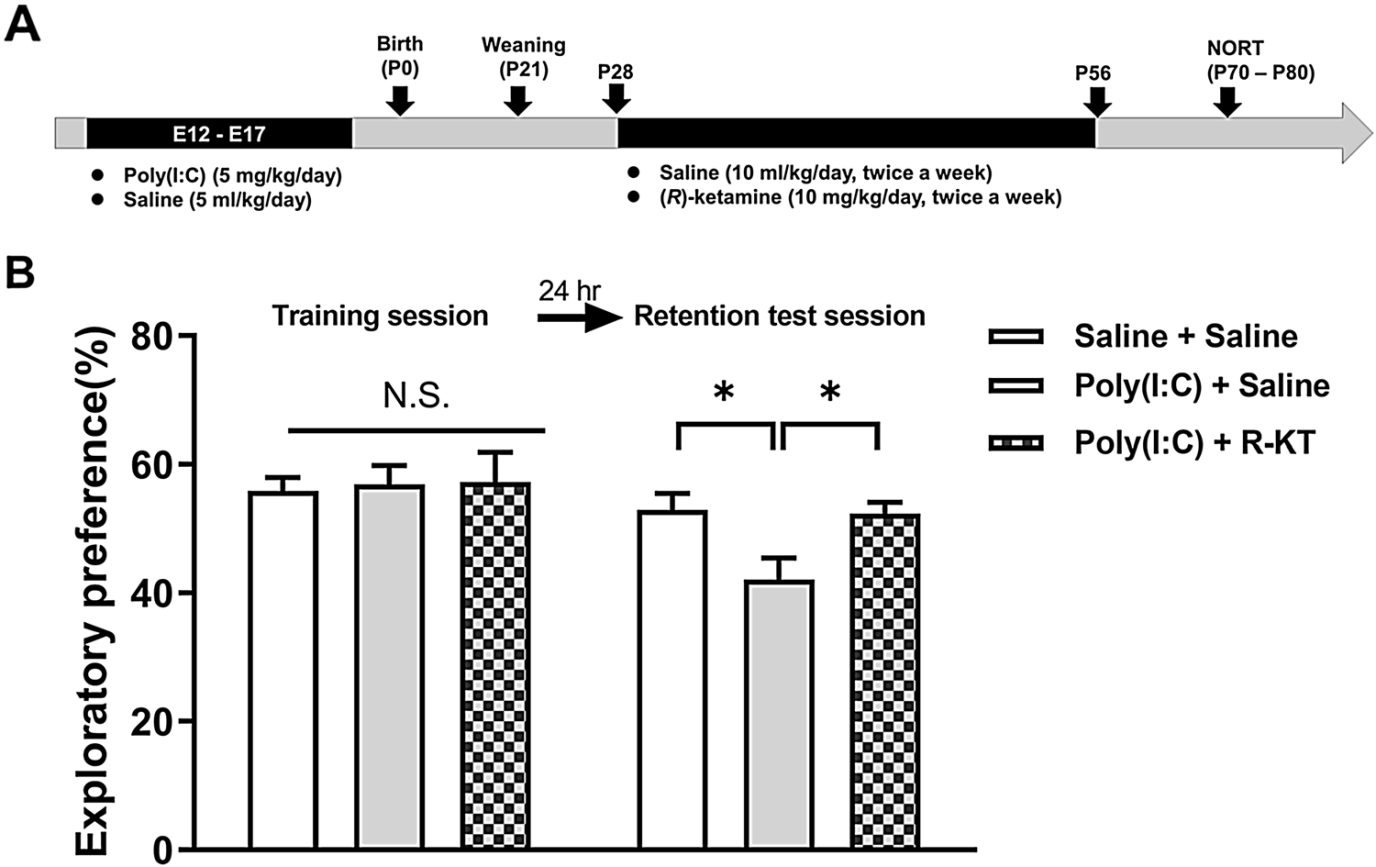
Effects of (*R*)-ketamine on cognitive deficits in adult offspring after prenatal poly(I:C) exposure. **A** Schedule of treatment and behavioral tests. Saline (5.0 ml/kg/day) or poly(I:C) (5.0 mg/kg/day from E12 to E17) was injected i.p. into pregnant mice. Male offspring was separated from mothers on P21. Saline (10 ml/kg/day, twice a week) or (*R*)-ketamine (10 mg/kg/day, twice a week) was administered i.p. to male offspring from P28 to P56. Novel object recognition test (NORT) was performed from P70-P80. Brain samples for PV-immunohistochemistry were collected at P80. **B** NORT: There was no difference between the three groups in the training session. In the retention session, the exploratory preference of poly(I:C) + saline group was significantly lower than vehicle + saline-treated group. *P < 0.01 compared with poly(I:C) + saline-treated group. N.S.: not significance. The value is expressed as the mean ± S.E.M. (*n* = 8)

Saline (10 ml/kg/day, twice a week, for 4 weeks) or (*R*)-ketamine (10 mg/kg/day as hydrochloride salt, twice a week, for 4 weeks) was administrated i.p. into male offspring (Fig. 1A). In the experiment using TrkB inhibitor ANA-12, the vehicle (10 ml/kg) or ANA-12 (0.5 mg/kg) was injected i.p. 30 min before saline (10 ml/kg/day) or (*R*)-ketamine (10 mg/kg/day) (Fig. 4A).

### Novel object recognition test (NORT)

To assess cognitive function in mice, NORT was performed as previously reported [18–21, 46, 55]. Each mouse was allowed to habituate in the field for 10 min a day to adapt to the experimental equipment for 3 consecutive days. During the training process, two novel objects (different in shape and color, but similar in size) were placed in a box 35.5 cm apart (symmetrically), and each mouse was allowed to explore freely in this open field for 10 min. When the mouse’s head touches or stands on an object, the mouse is considered to be exploring the object. The time the mouse explored each object was recorded. After the training, the mice were immediately returned to their cages, and the boxes and objects were washed with 75% ethanol to avoid any possible instinctive odor cues. 24 h after training, the retention test was performed in the same box, and one of the familiar objects was replaced by a new object during the retention session. Then let the mice explore freely for 5 min and record the time spent exploring each object. Throughout the experiment, these objects are balanced in terms of physical complexity and emotional neutrality. The preference index, which is the ratio of the time spent exploring two objects (training session) or a new object (retention test session) to the total time spent exploring two objects, is used to measure recognition memory.

### PV-immunohistochemistry

Immunohistochemistry of PV in the mouse brain was performed, as previously reported [19, 21]. Mice were deeply anesthetized with pentobarbital sodium, and 10 mL of isotonic saline was added, followed by 40 mL of cold 4% paraformaldehyde 0.1 M phosphate buffer (pH 7.4). The brain was removed from the skull and immobilized overnight at 4 °C in the same fixator. For immunohistochemical analysis, continuous sections of coronal brain tissue 50 μm thick were cut using a vibrating blade micro slicer (VT1000S, Leica Microsystems AG, Wetzlar, Germany) in a cold 0.01 M phosphate buffer (pH 7.5). Free-floating sections were treated with 0.3% H_2_O_2_ in 50 mM Tris–HCl saline (TBS) for 30 min and sealed in TBS containing 0.2% Triton X-100 (TBST) and 1.5% normal goat serum for 1 h at room temperature. The sample was then incubated with rabbit polyclonal anti-PV antibodies (1:2,500, Swant, Bellinzona, Switzerland) at 4 °C for 24 h. Sections were washed three times in TBS, and then the avidin–biotin-peroxidase method was used (Vectastain Elite ABC, Vector Laboratories, Inc., Burlingame, CA, USA). Sections were incubated in 0.25 mg/mL diaminobenzidine solution containing 0.01% H_2_O_2_ for 3 min. The slides were then installed on the gelatinized glass slide, dehydrated, cleaned, and covered with cover glass (Fisher Scientific, Fair Lawn, NJ, USA). The sections were imaged, and the staining intensity of PV-immunoreactivity in the infralimbic (IL) and prelimbic (PrL) of the medial prefrontal cortex (mPFC), and CA1, CA3 and dentate gyrus (DG) of hippocampus was analyzed using an optical microscope equipped with a CCD camera (Olympus IX70, Tokyo, Japan) and the SCION IMAGE software package. Images of sections within brain regions were captured using a 100 × objective with a Keyence BZ-9000 Generation II microscope (Osaka, Japan).

### Golgi staining

For Golgi staining in the brain, FD Rapid GolgiStain™ Kit (FD Neuro Technologies, Inc., Columbia, MD) was used as reported previously [23, 54, 56, 57]. The mice were deeply anesthetized with sodium pentobarbital. The brain was removed from the skull and rinsed with double distilled water. Golgi staining of the brain and preparation of brain sections were performed at Biopathology Institute Co., Ltd (Kunisaki, Oita, Japan). Brain regions such as mPFC, CA1, CA3, DG of hippocampus were taken under a Keyence BZ-9000 Generation II Microscope (Osaka, Japan) with a 100 × objective lens. The number of dendritic spine density in the brain regions was counted. For spine density measurement, the clear and assessable areas were used, including the 50–100 μm of the secondary dendrites of each imaged neuron. To determine the relative spine densities, we counted the number of spines of multiple dendritic branches of a single neuron to obtain the average number of ranges per 10 μm. For the number of spines measured, only those spines that appear perpendicular to the dendritic axis are counted. We analyzed 3 parts of each neuron and 3 parts of each animal.

### Statistical analysis

The data are shown as the mean ± standard error of the mean (SEM). The analysis was performed using PASW Statistics 20 (formerly SPSS Statistics; SPSS, Tokyo, Japan). Data including the NORT, immunohistochemistry results, and dendritic spine density, were analyzed by one-way analysis of variance (ANOVA), followed by *post-hoc* Tukey HSD test. A *P* value of less than 0.05 was considered statistically significant.

## Results

### Prophylactic effects of (R)-ketamine on cognitive deficits in adult offspring of prenatal exposure of poly(I:C)

In NORT, the repeated administration of poly(I:C) (5 mg/kg/day for 6 days) during E12 to E17 caused cognitive deficits in adult offspring (Fig. 1B). In the training session, there was no difference (one-way ANOVA: *F*_2,21_ = 0.040, *P* = 0.961) among the three groups (Fig. 1B). In the retention test session, there was a significant difference (one-way ANOVA: *F*_2,21_ = 5.330, *P* < 0.05) among the three groups. The repeated intermittent administration of (*R*)-ketamine (10 mg/kg/day, twice a week, 4 weeks) significantly ameliorated the decreased exploratory preference in adult offspring after prenatal poly(I:C) exposure (Fig. 1B).

### Effects of (R)-ketamine on decreased PV-immunoreactivity in the mPFC of adult offspring after prenatal poly(I:C) exposure

**Fig. 2:**
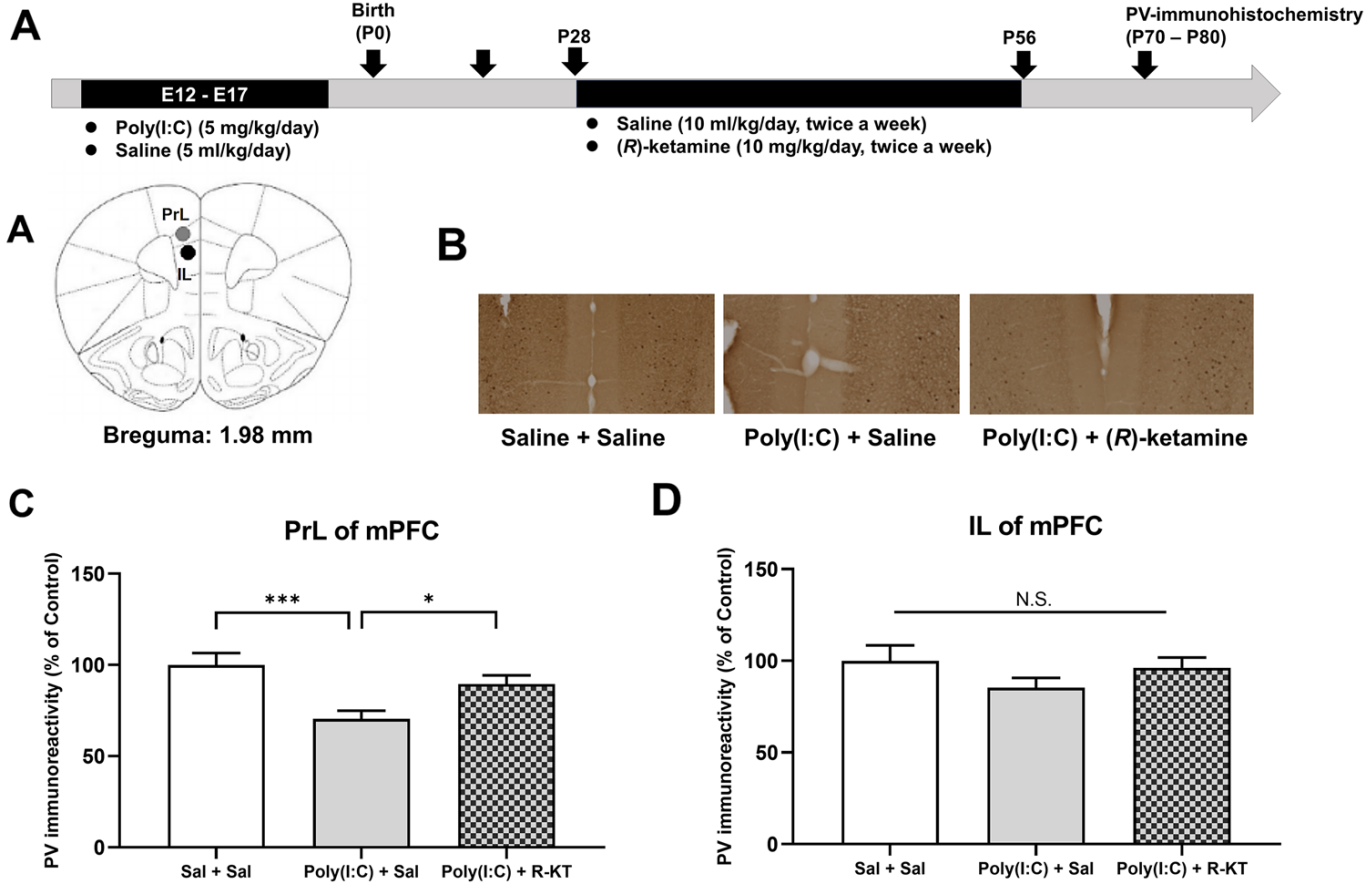
Effects of (*R*)-ketamine on decreased PV-immunoreactivity in the mPFC of adult offspring after prenatal poly(I:C) exposure. **A** Brain atlas of prelimbic (PrL) area and infralimbic (IL) area of the medial prefrontal cortex (mPFC). **B** Representative data of PV-immunoreactivity in the mPFC of adult offspring after MIA. **C** The PV-immunoreactivity in the PrL of mPFC of poly(I:C) + saline-treated group was significantly lower than that of other two groups. **D** There were no changes for PV-immunoreactivity in the IL among the three groups. *P < 0.05, ***P < 0.001, compared with poly(I:C) + saline-treated group. N.S.: not significance. The value is expressed as the mean ± S.E.M. (*n* = 10)

We previously reported that reduction of PV-positive cells in the mPFC might be associated with cognitive deficits in offspring after MIA [19]. We performed PV-immunohistochemistry in the mPFC from adult offspring after MIA (Fig. 2A). One-way ANOVA analysis revealed significant effects (PrL: *F*_2,27_ = 7.927, *P* < 0.001) among the three groups (Fig. 2C). The *post-hoc* test showed that PV-immunoreactivity in the PrL of mPFC of poly(I:C) + saline group was significantly lower than that of vehicle + vehicle group or poly(I:C) + (*R*)-ketamine groups (Fig. 2C). In contrast, one-way ANOVA analysis of IL data revealed no significant effects (IL: *F*_2,27_ = 1.311, *P* = 0.227) among the three groups (Fig. 2D). These findings suggest that adult offspring after prenatal poly(I:C) exposure showed the loss of PV-immunoreactivity in the PrL of mPFC, but not IL, and that repeated intermittent administration of (*R*)-ketamine could prevent the loss of PV-immunoreactivity in the PrL of mPFC of adult offspring after prenatal poly(I:C) exposure.

### Effects of (R)-ketamine on dendritic spine density in the brain of adult offspring after prenatal poly(I:C) exposure

**Fig. 3:**
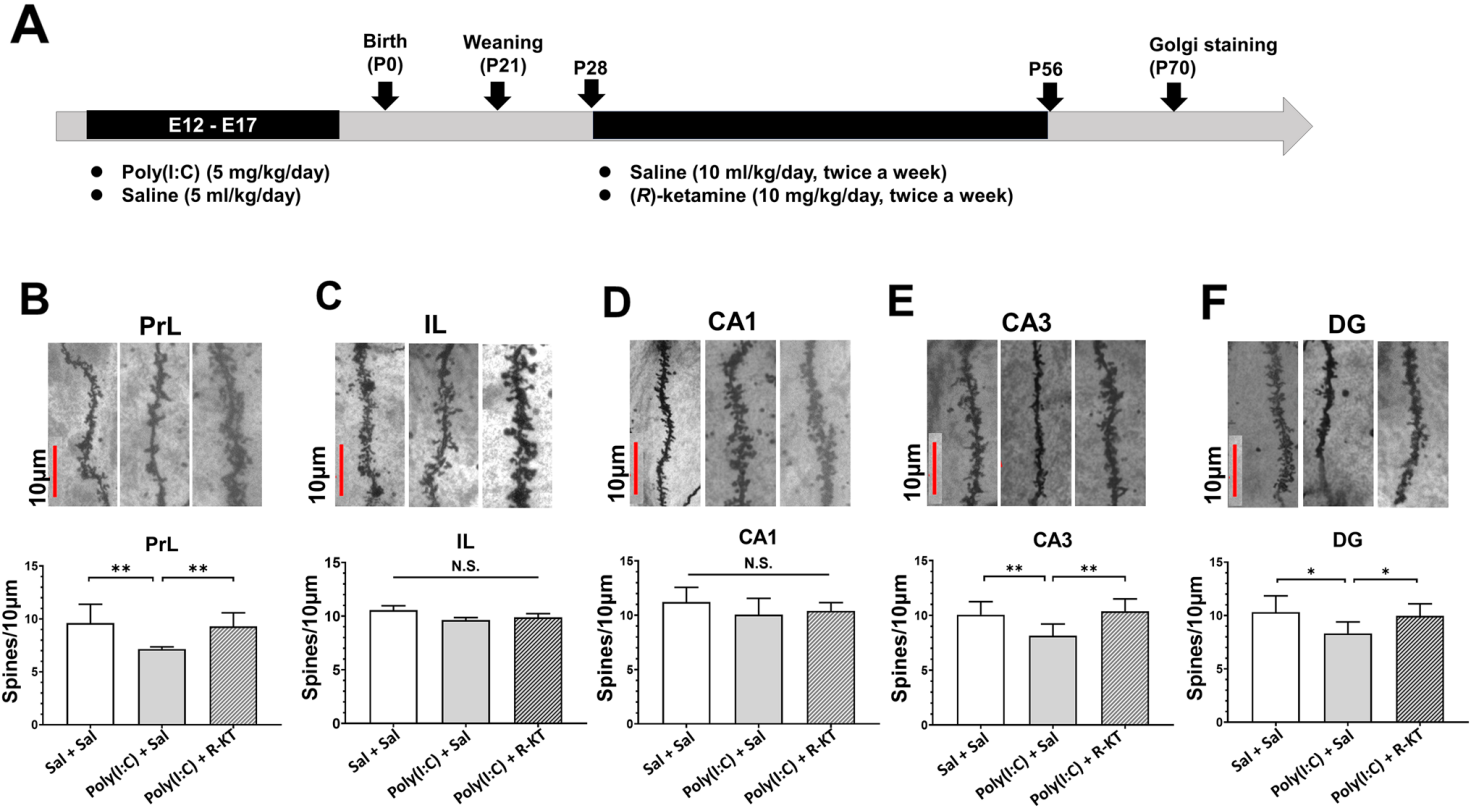
Effects of (*R*)-ketamine on dendritic spine density in the brain of adult offspring after prenatal poly(I:C) exposure. **A** Schedule of treatment and behavioral tests. Saline (5.0 ml/kg/day) or poly(I:C) (5.0 mg/kg/day from E12 to E17) was injected i.p. into pregnant mice. Male offspring was separated from mothers on P21. Saline (10 ml/kg/day, twice a week) or (*R*)-ketamine (10 mg/kg/day, twice a week) was administered i.p. to male offspring from P28 to P56. Brain samples for Golgi staining were collected at P70. **B**–**F** Representative images of Golgi staining in the mPFC and hippocampus of adult offspring after MIA. The number of dendritic spine density in the PrL of mPFC, CA3, and DG of poly(I:C) + saline-treated group was significantly lower than that of the other two groups. In contrast, there were no changes for spine density in the PL and CA1 among the three groups. *P < 0.05, compared with poly(I:C) + saline group. **P < 0.01, compared with poly(I:C) + saline group. N.S.: not significance. The value is expressed as the mean ± S.E.M. (*n* = 8)

Dendritic spine density measurement was performed in adulthood offspring after MIA. One-way ANOVA analysis revealed that dendritic spine densities in the PrL region of the mPFC, CA3 and DG regions of the hippocampus from adult offspring after prenatal poly(I:C) exposure were statistically significant (PrL: *F*_2,21_ = 22.97, *P* < 0.001; CA3: *F*_2,21_ = 23.80, *P* < 0.001; DG: *F*_2,21_ = 12.22, *P* < 0.001) among the three groups (Fig. 3B, E, F). The *post-hoc* test showed that dendritic spine density in the PrL of mPFC, CA3 and DG of the hippocampus from poly(I:C) + saline group was significantly lower than that of saline + saline group or poly(I:C) + (*R*)-ketamine group (Fig. 3B, E, F). In contrast, one-way ANOVA analysis revealed that dendritic spine densities in the IL regions of the mPFC, CA1 region of the hippocampus from the adult offspring after prenatal poly(I:C) exposure were not statistically significant (IL: *F*_2,21_ = 0.1132, *P* = 0.894; CA1: *F*_2,21_ = 2.829, *P* = 0.082) among the three group (Fig. 3C, D). These findings suggest that adult offspring after prenatal poly(I:C) exposure showed the decreased spine density in the PrL of mPFC, CA3 and DG of the hippocampus, and that repeated intermittent administration of (*R*)-ketamine could ameliorate the decreased spine density in these brain regions after MIA.

### Effects of ANA-12 in the effects of (R)-ketamine on cognitive deficits in adult offspring after prenatal poly(I:C) exposure

**Fig. 4:**
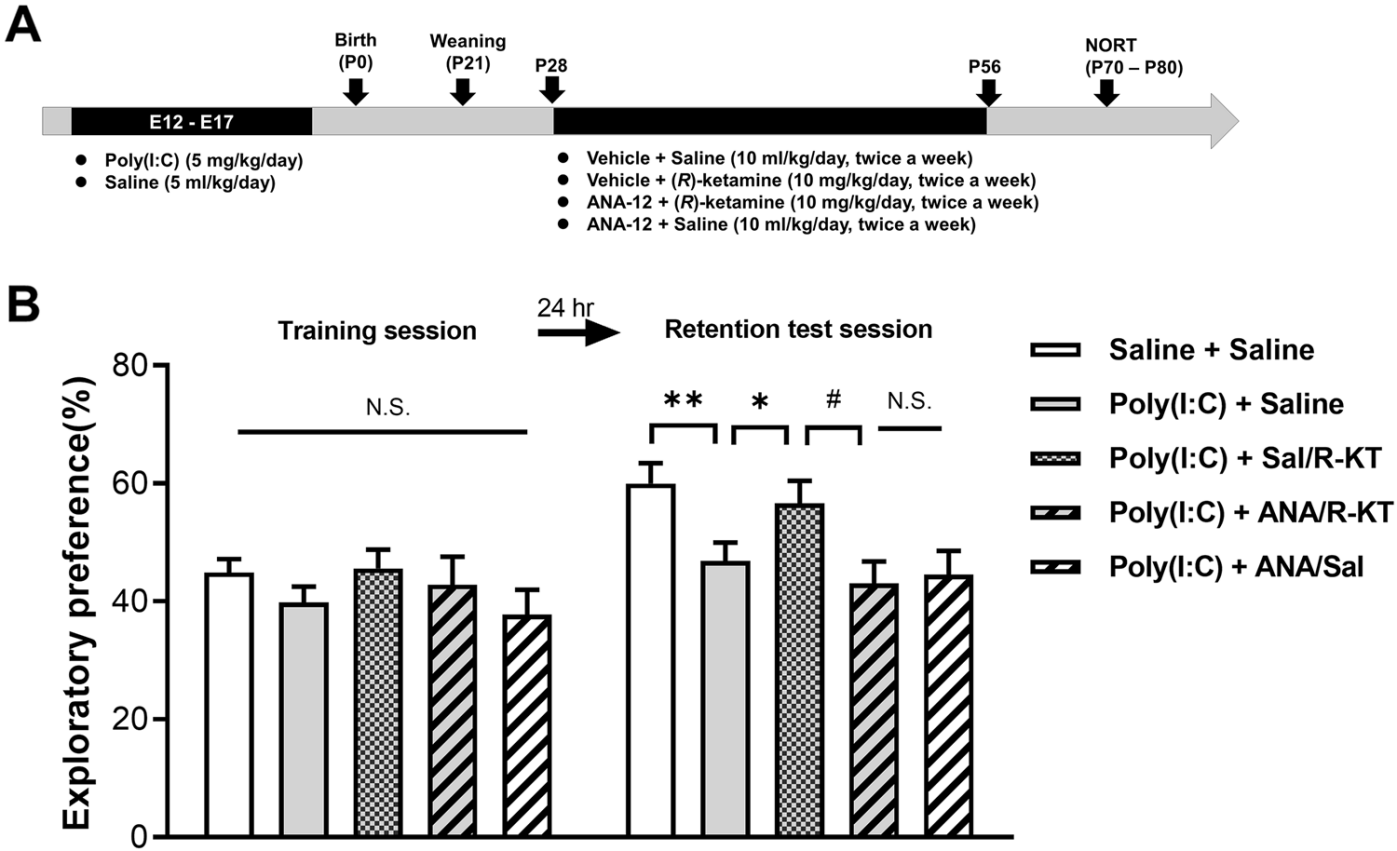
Effects of ANA-12 on prophylactic effects of (*R*)-ketamine on cognitive deficits in adult offspring after prenatal poly(I:C) exposure. A: Schedule of treatment and behavioral tests. Saline (5.0 ml/kg/day) or poly(I:C) (5.0 mg/kg/day from E12 to E17) was injected i.p. into pregnant mice. Male offspring was separated from mothers on P21. Saline (10 ml/kg/day, twice a week) or (*R*)-ketamine (10 mg/kg/day, twice a week) was administered i.p. to male offspring from P28 to P56. Vehicle (10 ml/kg/day) or ANA-12 (0.5 mg/kg/day) was injected i.p. 30 min before injection of saline or (*R*)-ketamine. Novel object recognition test (NORT) was performed from P70-P80. B: NORT: There was no difference between the five groups in the training session. In the retention test session, the exploratory preference of poly(I:C) + vehicle/saline group was significantly lower than saline + vehicle /(*R*)-ketamine group. The exploratory preference of poly(I:C) + vehicle/(*R*)-ketamine group was significantly higher than poly(I:C) + ANA/(*R*)-ketamine group. *P < 0.05, **P < 0.01 compared to poly(I:C) + vehicle/saline group. ^#^*P* < 0.05 compared to poly(I:C) + vehicle/(*R*)-ketamine group. N.S.: not significance. The values were expressed as the mean ± S.E.M. (*n* = 7–14)

To examine the role of TrkB signaling pathway in the prophylactic action of (*R*)-ketamine, we investigated the effects of the TrkB inhibitor ANA-12 in the juvenile offspring of prenatal mice exposed to poly(I:C) (Fig. 4A). In the training session, there were no significant differences (one-way ANOVA: *F*_4,52_ = 0.9507, *P* = 0.442) between the five groups (Fig. 4B). In the retention test session, there were significant differences (one-way ANOVA: *F*_4,52_ = 4.305, *P* < 0.01) between the five groups (Fig. 4B). The pretreatment with ANA-12 significantly antagonized the beneficial effects of (*R*)-ketamine in the offspring of prenatal mice exposed to poly(I:C) (Fig. 4B). In contrast, ANA-12 alone did not improve MIA-induced cognitive deficits (Fig. 4B).

## Discussion

The major findings of the present study are as follows. Repeated intermittent administration of (*R*)-ketamine during the juvenile and adolescent stages could prevent the development of cognitive deficits, reduced PV-immunoreactivity in the PrL of mPFC and reduced dendritic spine density in the PrL of mPFC, CA3 and DG of the hippocampus of adult offspring after prenatal poly(I:C) exposure. Furthermore, pretreatment with ANA-12 blocked the beneficial effects of (*R*)-ketamine in this model. These data suggest that (*R*)-ketamine shows prophylactic effects in MIA model through TrkB activation. Therefore, it is likely that treatment with (*R*)-ketamine in subjects with ultra-high risk for psychosis might block the subsequent conversion to psychosis in young adulthood.

We found cognitive deficits of juvenile offspring in mice after prenatal poly(I:C) exposure [18–21], indicating cognitive deficits as prodromal symptoms. We reported that supplementation with TrkB receptor agonist 7,8-dihydroxyflavone (1 mg/mL in drinking water) during juvenile and adolescent stages could prevent cognitive deficits as well as reduced BDNF-TrkB signaling in the PFC of adult offspring after MIA [19]. In this study, we found that pretreatment with ANA-12 blocked the prophylactic effects of (*R*)-ketamine in MIA-induced model. Collectively, it is likely that (*R*)-ketamine can exert beneficial effects by activating BDNF-TrkB signaling in the brain.

(*R,S*)-ketamine has been used widely as an animal model of schizophrenia since (*R,S*)-ketamine is reported to cause schizophrenia-like symptoms (i.e., positive and negative symptoms, and cognitive impairment) in healthy control subjects [58, 59]. In contrast, a recent systematic review showed that subanesthetic dose of (*R,S*)-ketamine (0.5 mg/kg) showed significant improvements in cognitive impairment in treatment-resistant patients with depression, although (*R,S*)-ketamine did not worsen cognitive function in depressed patients [41, 60]. Thus, it seems that the effects of (*R,S*)-ketamine on the cognition of healthy control subjects and patients with psychiatric disorders are not consistent. Importantly, it is reported that (*R*)-ketamine has less detrimental side effects than (*R,S*)-ketamine or (*S*)-ketamine in humans [33, 34, 61]. Preclinical data show that abuse liability of (*R,S*)-ketamine in humans is primarily due to the pharmacological effects of (*S*)-ketamine, but not (*R*)-ketamine [23, 27, 35]. Thus, it seems that (*R*)-ketamine is safer than (*R,S*)-ketamine or (*S*)-ketamine in humans. We recently reported that PCP-induced cognitive deficits in mice were ameliorated by subsequent repeated intermittent administration of (*R*)-ketamine (10 mg/kg/day, twice weekly for 2-weeks), but not (*S*)-ketamine [46]. Taken together, it is likely that (*R*)-ketamine would be a potential prophylactic drug for schizophrenia when it can be used in subjects with high-risk for psychosis.

It is suggested that the decrease of PV-positive cells in the PFC may be related to the pathogenesis of schizophrenia [62, 63]. Study using postmortem brain samples showed that dendritic spine density in PFC of patients with schizophrenia was decreased compared with controls [64]. Previous studies have shown that prenatal poly(I:C) exposure altered dendritic morphology in the PFC of non-human primate MIA models [65]. In this study, we found that the reduced PV-immunoreactivity in the PrL of mPFC and reduced dendritic spine density of PrL of mPFC, CA3 and DG of the hippocampus from adult offspring after MIA was significantly ameliorated by subsequent repeated intermittent administration of (*R*)-ketamine (10 mg/kg/day). Given the role of mPFC in cognitive function, it is noteworthy that (*R*)-ketamine could improve decreased PV-immunoreactivity and dendritic spine density in the mPFC of offspring after MIA.

Depression is common in subjects at ultra-high risk for psychosis, and associated with extensive functional impairment [66]. A longitudinal study showed that persistent negative symptoms exist in youth at clinical high risk for psychosis, resulting in significant and persistent functional impairment [67]. Considering the therapeutic potential of (*R*)-ketamine in depression, it is likely that (*R*)-ketamine could be a therapeutic drug for depression in subjects at high risk for psychosis, resulting in improvement of functional impairment.

In conclusion, the present data suggest that repeated intermittent administration of (*R*)-ketamine during juvenile and adolescent stages could prevent the onset of cognitive deficits and the loss of PV-immunoreactivity in the PrL of mPFC, the decreased spine density in the PrL of mPFC, CA3 and DG of hippocampus from adult offspring after MIA. Therefore, it is possible that the use of (*R*)-ketamine in young subjects at high risk for psychosis may prevent conversion to psychosis from prodromal symptoms.
